# Black women and COVID-19: The need for targeted mental health research and practice

**DOI:** 10.1177/1473325020973349

**Published:** 2021-03

**Authors:** Quenette L Walton, Rosalyn Denise Campbell, Joan M Blakey

**Affiliations:** Graduate College of Social Work, University of Houston, Houston, USA; School of Social Work, University of Georgia, Athens, USA; School of Social Work, Tulane University, New Orleans, USA

**Keywords:** COVID-19, Black women, mental health, social work research, and practice

## Abstract

COVID-19 has significantly impacted a substantial number of Black Americans. Black women, in particular, are facing challenges financially, physically, and mentally during this unprecedented time. Between serving as frontline workers, being concerned about contracting the virus, contributing to their families financially, and worrying about their loved ones’ health, Black women are experiencing great strain on their mental health and well-being. These stressors illustrate the need for social work researchers and practitioners to address Black women’s mental health. This paper presents our reflections, experiences, and response to COVID-19 as Black women and scholars. Guided by our reflections and personal experiences, we put forth suggestions and reflexive thoughts for social work researchers and practitioners to prioritize Black women’s mental health during and after these unprecedented times.

COVID-19 has had a devastating impact on the health and well-being of individuals and communities across the globe. As of October 2020, over seven million COVID-19 cases, over 300,000 new cases, and over 200,000 people have died from COVID-19 related complications in the United States (Centers for Disease Control (CDC), 2020; Jacobson et al., 2020). The pandemic has led to increased isolation due to social distancing, stay-at-home orders, and voluntary and involuntary quarantines. It continues to overwhelm health care systems’ capacities and capabilities. It has exhausted the patience and resolve of leaders and citizens trying to slow down the spreading of the virus. COVID-19 also has exacerbated existing health disparities and revealed a gap in the United States national, local, and county governments’ ability to respond to our most vulnerable populations’ health needs, especially Black people who account for most deaths across all age categories (Ford et al., 2020). These abrupt changes in daily living, compounded by witnessing the loss of family members and friends due to this virus, have taken a significant toll on Black women’s psyche.

Given the increased awareness of the impact that COVID-19 has on Black Americans, social work researchers and practitioners have a tremendous opportunity to privilege the mental health needs of Black women. This paper presents our reflections, experiences, and response to COVID-19 as Black women scholars. Guided by our reflections and personal experiences, we offer suggestions for current and future social work researchers and practitioners to prioritize Black women’s mental health during and after these unprecedented times.

## Positionality

As Black women, scholars, and qualitative researchers observing COVID-19 ravish our country and communities, we saw the inevitable coming. Our lived experiences and research highlighted the impact health disparities and racism would have on Black women’s mental health. Given what we know about health disparities, which existed long before the pandemic, we predicted devastating outcomes for Black Americans. We also knew what all of this would mean for us as Black women scholars: not only would our cultural knowledge and culturally-responsive skills be tasked and taxed to explain once again “why Black folks suffer,” but we would also have to watch, in real time, as these issues we study play out in our communities and our homes. It is times like these, in this fight against COVID-19, that our identities as Black women scholars exist as both personal and professional. 

Professionally, we know that we must respond to and help mitigate the impact of the pandemic on Black people’s lives or soothe those who COVID-19 has already injured and scarred. However, the responsibility to do so must be a shared one. As Black women, scholars, and qualitative researchers, we can no longer, no matter how willing or how “strong,” shoulder this task alone. As social workers, we speak at length about not using the profession to “heal thyself.” Yet, Black women are often called upon to heal those around us and then ourselves. When we fail to support Black women, we intensify and exacerbate the culture of violence based on race and gender that has become normalized in the United States (Perry, 2020). Support for Black women in academia should be just as diverse as the Black women in these institutions and must include opportunities for funding, leadership, and space to thrive and be authentically ourselves.

## Black Women and Mental Health

Black Americans are contracting the coronavirus at higher rates (Ford et al., 2020). Systemic racism places Black women at higher incidences of underlying health conditions like hypertension, diabetes, and breathing issues, which make Black Americans much more susceptible to contracting the virus and experiencing longer and more complicated courses of the illness and death (Bailey et al., 2020; Bailey & Moon, 2020; Reyes et al., 2020).

Black women are experiencing financial and mental angst directly related to the COVID-19 pandemic. Systemic racism and its intersection with other systems of oppression are underlying factors that place Black women in employment and caregiving positions that increase their exposure to the deadly virus. Financially, Black women are more likely to be in positions where they do not have the financial reserves to cover expenses during an emergency like COVID-19 (Lopez et al., 2020). Once Black women contract the disease, they are more likely to die because they often do not have access to affordable healthcare (Reyes et al., 2020).

The toll COVID-19 has on Black women’s psychological well-being cannot be underestimated or overlooked. There are high levels of stress, anxiety, depression, generalized fear, pervasive feelings of hopelessness and desperation, and suicidal behavior associated with mass quarantine, isolation, and ongoing uncertainty regarding the future (Serafini et al., 2020; Xiang et al., 2020). As the financial impacts related to COVID-19 become more pronounced, the psychiatric conditions will likely become more pervasive leading to community anxiety and collective hysteria (Lee, 2020; Serafin et al., 2020). Mentally, Black women have to put on the culturally dictated “strong face” despite concerns about contracting the virus, contributing to our families financially, and worrying about loved ones’ health. The compounded effects of these additional financial, physical, and mental health burdens create a perfect storm that demands our full attention.

When reviewing all that Black women have sacrificed and contributed, researchers and practitioners must understand and privilege Black women’s needs and experiences during and after the COVID-19 pandemic (Belgrave & Allison, 2018). Social work purports to work with the most vulnerable, marginalized, and oppressed populations. Thus, social work researchers and practitioners are in a unique position to respond to the mental health needs and experiences of Black women during this unprecedented pandemic.

## The Need for Social Work Researchers and Practitioners

Contemplating our own experiences during COVID-19 and recognizing the parallels with other Black women’s experiences, we are compelled to offer some recommendations to the current and future social work professionals around engaging and intervening with Black women during and after this pandemic. Through the exploration of our collective coronavirus narratives as Black women, we noted three crucial aspects (i.e., sociocultural context, training, and leadership) related to Black women’s mental health that social workers need to understand.

### Sociocultural Context

Context matters! We cannot say this enough; as Black women, we are living with unimaginable stress. The need to address the historical impact of racism and discrimination in the United States is vitally important. Black women were struggling before COVID-19. This pandemic only exacerbates the disparities and stressors for Black women. It is essential to understand the chronic psychological stress associated with the intersecting identities of being Black and a woman (Simien, 2020). As mental health social work researchers and practitioners, we have to create the space and the opportunity for Black women, living during the tumultuous times of COVID-19, to articulate their standpoint and value their subjective knowledge (Collins, 2000). Doing so will provide a different narrative that explicitly considers and integrates the sociocultural context in which Black women live. It also will show the importance of prioritizing Black women’s mental health and promoting practices that privilege their lived experiences and voices resulting from the impacts related to COVID-19. However, to do so, current and future social work researchers and practitioners have to think about Black women’s sociocultural context explicitly.

### Training

Research shows that social workers provide most of the United States mental health services. This number is expected to increase over the years (Bureau of Labor Statistics, 2020; Council on Social Work Education, 2014; Heisler, 2018). The Bureau of Labor Statistics (2020) noted that as mental health needs increase in the United States, the need for mental health social workers is expected to grow by 18% by 2028. The mental health needs of Black women before COVID-19 were increasing steadily; COVID-19 will only exacerbate them (Simien, 2020). Social workers must be able and be equipped to fulfill these roles effectively.

An important challenge for the social work profession is to avoid replicating the racist, patriarchal practices that have created trauma and mistrust for Black women engaged in mental health services and research in addition to embracing this devastating pandemic. We must also ensure that social workers are equipped to investigate, inform, and provide essential mental health resources to Black women using a sociocultural lens. Additionally, schools of social work must teach students to work with Black women effectively by providing them with field placements and incorporating articles, books, and other materials into the curriculum written by Black women. To effectively address Black women’s unique mental health needs, schools of social work must hire Black faculty who have experience working and conducting research with Black women. Further, we can focus more intentionally on our Code of Ethics by specifically paying attention to the call for training to be sensitive to cultural and ethnic diversity, and for making a commitment to end discrimination, oppression, and other forms of social injustice (National Association of Social Workers (NASW), 2017). As researchers and practitioners, we should use social work values and ethical practices as a guide to ensure we are understanding and addressing the unique needs the COVID-19 pandemic has created for Black women in terms of their lived experiences with discrimination and oppression.

### Leadership

A message often heard or expressed colloquially, whether lamenting about national leadership blunders or disparaging friends’ cultural missteps, is “[you should] listen to Black women.” Although expressed jokingly, there is great truth to the statement. Our lived experiences as Black women have not only made us keenly aware of what is problematic across many socio-political and cultural landscapes, but they also have made us more resourceful and creative when addressing complex issues, like COVID-19. As Black women scholars in social work, we frequently share stories about how colleagues often extract experiential knowledge from the stories of our lives, and we applaud their use, but typically only when this knowledge informs what we “do” as social workers. Whether it is how we mentor or support a struggling student or recognize how intensely we problem-solve and advocate for our clients, our colleagues recognize our labor. What is recognized far less, if at all, is how the depth and breadth of our experiences inform and contribute to our timely and novel research or shape and inspire our visions of social work education and practice.

It is time for the social work profession, especially during this time of great turmoil, to recognize Black women directly and explicitly for the leadership and guidance we provide when addressing the widespread issues brought on by COVID-19. We do not mean viewing us as cultural informants or behind-the-scenes confidants to existing leaders; we mean recognizing our value to be invited to “the table,” trusted enough to restructure “the table,” and respected enough to turn “the table” over if need be. Much in the same way our profession seeks to cease replicating patriarchal and racist structures in how we work with our clients, we need to do the same as colleagues. We can accomplish this by acknowledging, respecting, and yielding to Black women’s leadership drawn from the knowledge, expertise, and skills borne out of our intersectional existence and experiences as Black women. So, as we approach addressing the mental health needs of Black women experiencing the disproportionate weight of COVID-19, we must rely on Black women researchers’ and practitioners’ experiences and knowledge to lead the charge in restoring, protecting, and promoting the mental health of already overburdened Black women.

## “Black Tax”

Black tax is a phrase in which Black people are expected to work harder and “in addition to the usual stresses, confront a set of personalized social strains which grow out of their “blackness” in a predominantly White environments (Harper, 1975, 207). With respect to Black women, even more of us is expected as Black people and women give significantly more than others, often without recognizing the sacrifices it requires us to make (Carter-Black, 2008).

- We are called upon to save everything, including the United States, come November 2020 for the consequential election (Perry, 2020).
- We are asked to save our students from the stress and weight of the coronavirus pandemic.
- We are asked to save our departments from the increased scrutiny around how we view, assess, and address diversity, equity, and inclusion.
- We are asked to save a random grant or a piece of scholarship that has failed to recognize the complex lives in which Black people live.
- We are asked to save our communities by lending our labor and voices to causes that sometimes undermine our well-being.
- We are asked to save our families from the impact of a world that does not see our lives as valuable or worthy.
- And we are tired!

Black women’s exhaustion does not mean we are unwilling or unable to address myriad issues. However, we are rarely provided the resources—financial, social, space, community—and the support (i.e., encouragement, validation, advocates) necessary to do so. We are asked to do more to advance others’ needs but then are routinely passed over for opportunities to advance our careers for promotion and tenure (Griffin et al., 2013). Our stories and the available empirical evidence suggest that the intersection of racism and sexism Black women *like us* experience in academia presents unique challenges that impact how we are perceived, how we are treated, and how we are evaluated as professors (Griffin et al., 2013; Pittman, 2010). So, when we—Black women—say what we need to be successful in academia or identify the resources we need to create the space for us to be successful, we are still not heard, or our work is called into question. Thus, addressing the many issues within our departments, schools, or colleges of social work still does not guarantee advancement for us. So, we are sick and tired of being sick and tired!

The field of social work as a whole must see Black people, and Black women in particular, as valuable assets. Until then, we no longer want to have conversations about why diversity efforts fall on deaf ears or why inclusion and equity platforms are needed to intentionally create and sustain social work because Black women’s work will never be done. We teach in these institutions, we create in these institutions, and we help sustain these institutions. Yet, the additional work we perform often remains unrecognized by schools and affects Black women’s ability to get tenure and promotion (Griffin et al., 2013).

Thus, we briefly discussed the three aforementioned factors (i.e., sociocultural context, training, and leadership) that social workers must consider when working with Black women who experience mental health challenges during COVID-19 because *Black women’s* mental health is in jeopardy. It is vital that we pay attention to and center the narratives of Black women who have been historically oppressed and continually marginalized. Now, it is the field’s (practitioners’ and researchers’) responsibility to address this charge—the mental health of Black women!

## Conclusion

We implore the social work profession to value Black women as scholars, not solely for our strength, endurance, and mental fortitude as researchers and practitioners, but also for our leadership and ability to engage members. As Black women scholars who conduct qualitative research, we see our role in addressing COVID-19 as more critical now than ever. The complex interplay between physical, financial, and psychological affects resulting from COVID-19 related complications are devastating the Black community (Dyer, 2020). As social work researchers and practitioners, we need to ask questions that qualitatively explore what happened instead of why things have happened. In order to get to the root of the problems that adversely contribute to Black women’s mental health, social work researchers and practitioners also need to have candid conversations about power, privilege, oppression, and their roles as gatekeepers. Otherwise, social work researchers and practitioners will fail to offer the bold solutions needed to address the legacy of structural racism that negatively impacts Black women’s mental health during and post-COVID-19.
